# Clinical variability of respiratory pulmonary hypertension: implications for diagnosis and management

**DOI:** 10.1186/2049-6958-8-72

**Published:** 2013-11-26

**Authors:** Lucio Casali, Pierluigi Carratù, Matteo Sofia

**Affiliations:** 1 Respiratory Diseases, University of Perugia, Perugia, Italy; 2 Institute of Pulmonary Diseases, University of Medicine, Bari, Italy; 3 Respiratory Diseases, Monaldi Hospital, University Federico II, Naples, Italy

**Keywords:** Chronic obstructive pulmonary disease, Idiopathic pulmonary fibrosis, Obesity hypoventilation syndrome, Obstructive sleep apnea, Phenotype, Pulmonary hypertension, Pulmonary arterial hypertension

## Abstract

Pulmonary Hypertension (PH) associated to chronic respiratory diseases is currently classified in the 3^rd^ group, as a mild to moderate form of pre-capillary PH that progressively complicates the prognosis of associated pulmonary disease. In clinical practice, however, some unresolved issues in the respiratory PH should be considered: 1) the PH heterogeneity in some respiratory diseases, such as Chronic Obstructive Pulmonary Disease (COPD), where the prevalence of unrecognized left heart disease, or chronic pulmonary thromboembolism may change the clinical classification; 2) the combining form of severe PH which often is not correlated to chronic ventilator impairment, while outcome is strictly related to pulmonary haemodynamics. The recognition of out of proportion respiratory PH in several chronic respiratory diseases which include COPD, Idiopathic Pulmonary Fibrosis (IPF), Combined Pulmonary Fibrosis and Emphysema, Obstructive Sleep Apnea (OSA), Obesity Hypoventilation Syndrome (OHS) may be important for a comprehensive clinical classification of severe respiratory PH, as well as, for the inclusion of these patients in randomized clinical trials on PH targeted therapy.

## In memoriam

To the memory of my beloved Master Matteo Sofia, Mentor and Life’s Guide, who left me suddenly alone. Yours forever, Pierluigi Carratù.

To the everlasting memory of Prof. Matteo Sofia, great friend and colleague. Lucio Casali.

## Review

Clinical disease classification is an important tool for physicians, in order to distinguish severity and to differently manage a same disorder that can be found in multiple clinical conditions; this is particularly true for pulmonary hypertension [1,2]. Pulmonary Hypertension (PH) is a relatively frequent pathophysiological and haemodynamic state that can be detected in almost 37 clinical syndrome comprising multiple clinical conditions, including chronic left heart disease and lung diseases. The immediate advantages of the updated PH clinical classification are the separation of pulmonary arterial hypertension (Gr 1) (a rare disease, with a currently available efficacious treatment in term of life-savings) from other forms of PH associated to various disorders (Gr 2 and Gr 3), where specific pharmacological treatments for PH are discouraged, while the best management is represented by the treatment of associated disease. Actually Pulmonary Arterial Hypertension (PAH) includes different forms that share a similar clinical picture and virtually identical pathological changes of the distal pulmonary arteries which may represent the right substrate for specific therapy. Furthermore, the clinical classification allows the recognition of chronic thromboembolic PH, the only form surgically treatable (Gr 4), and a miscellaneous group of PH with unclear and/or multifactorial mechanisms (Gr 5) that may induce specialists to seek for important associations, such as ematologic, metabolic, or systemic diseases. Recently, the current 6 groups classification has given the opportunity to adhere to an easy stepwise diagnostic algorithm. There are, however, some limitations for clinical classification, especially when there is an inherent non homogeneous epidemiologic distribution with a high prevalence (85% of all PH cases) of Gr 2 and Gr 3 PH, compared to the other groups. It is likely that improving the concentration of PH in a same group may increase the clinical variability with differences in PH phenotype, making clinical evaluation more difficult for the greater interference of confounding factors related to comorbidities, such as age, increasing number of mixed forms, or a grey zone related to the selection of pulmonary haemodinamics criteria. Remarkably, this phenomenon has been reported also for PAH in the recent experience of US and UK registries. Compared to other analyzed comorbidities in PAH patients, hypertension, obesity, diabetes, and Chronic Obstructive Pulmonary Disease (COPD) were associated with significantly worse 6-minute walk distance (6MWD); obesity and COPD with worse functional class; and diabetes and COPD with increased risk of death [3]. Likewise, a grey zone of patients with Pulmonary Capillary Wedge Pressure (PCWP) 16 to 18 mm Hg who were diagnosed and treated for PAH has been analyzed: they were older, heavyweight, and more likely to have comorbidities associated with left ventricular diastolic dysfunction at diagnosis than those with PCWP ≤ 15 mm Hg. Five-year survival rates were similarly low for all PCWP subgroups [4]. Another report examined the influence of age on phenotypes of incident pulmonary arterial hypertension and showed the changes in demographics and epidemiology over the past decade in a national setting, suggesting that there may be two subtypes of patients: the youngest subtype with more severe hemodynamic impairment but better survival, and the oldest subtype which has more comorbidities [5].

The impact of coexistent left heart disease in COPD should be taken into account in clinical practice, as cardiovascular disease increases morbidity and mortality significantly. In one recent study, transthoracic echocardiography performed prospectively in more than 300 patients with COPD 3 months after discharge from the hospital after an acute exacerbation demonstrated significant cardiac alterations in 64% of patients: 27% left- and 48% right-heart disorders with a frequency of PH around 19%. Interestingly, left ventricle systolic dysfunction, left ventricle diastolic impairment, and left atrial dilatation were present in 29%, 12%, and 13% of the COPD patients respectively. Noticeably, echocardiographic abnormalities were unrelated to COPD severity [6]. The prevalence and the prognostic implications of the simultaneity of left ventricular dysfunction in COPD patients and airway obstruction in Congestive Heart Failure (CHF) patients were shown in another recent prospective study which included stable ≥ 60-yr-old patients with echocardiographic confirmed CHF (n = 201) and stable ≥ 60-yr-old patients with clinically and spirometry-confirmed COPD (n = 218). Previously unrecognized left ventricular dysfunction occurred in 17% of COPD with a significant influence on the survival rate [7].

The coexistence of emphysema and pulmonary fibrosis in the same patient results in a clinical syndrome, known as combined pulmonary fibrosis and emphysema (CPFE), that is characterized by dyspnea, upper-lobe emphysema, lower-lobe fibrosis, and abnormalities of gas exchange almost only in male older smokers. This syndrome is frequently complicated by pulmonary hypertension with increased mortality [8].

A retrospective multicentre study in 40 patients reported severe PH in CPFE patients and 60% survival at one year. Surprisingly, lung volumes and flows were relatively preserved while exercise capacity resulted severely compromised; moreover, haemodynamics were the most significant prognostic indexes [9].

PH is a well-recognized complication of interstitial lung disease, including idiopathic pulmonary fibrosis (IPF) and its prevalence has been estimated from 32% to 85%. There is a highly significant difference between survival of IPF patients with and without PH [10]. Hypoxia, destruction of pulmonary vascular bed or fibroproliferative phenomena might be important mechanisms in individual patients. However, the correlation of PH with forced vital capacity (FVC) is weak and patients with severe PH and still preserved lung volumes could also be seen [11]. Severe pulmonary hypertension can occur in idiopathic NSIP, even in the absence of advanced radiographic changes, especially when DL_CO_ is severely reduced [12].

Out-of-proportion PH is defined as the unjustified increase in PH that is observed in patients affected by different types of parenchymal lung disease [COPD, IPF, Obstructive Sleep Apnea, (OSA), Obesity Hypoventilation Syndrome (OHS), etc.]. The term ‘out-of-proportion PH’ was introduced recently into the field of PH. Until few years ago the term ‘pulmonary heart’ was used to indicate the development of PH-correlated parenchymal diseases and presence of chronic hypoxia leading to chronic respiratory failure and, as a consequence, right heart failure. But in the last few years better understanding of the mechanisms underlying structural remodeling of the pulmonary vascular bed raised the doubt that all can be explained by the presence of pulmonary heart. Indeed, some patients can develop extremely high pulmonary artery pressure that cannot be explained by hypoxia and remodeling of the pulmonary vascular bed alone. Due to an increasing use of right heart catheterization for selection of candidates for lung transplantation, a number of patients affected by parenchymal lung diseases (often accompanied by minor lung impairment on lung function tests and/or CT) with a not easily explainable degree of PH have come to the attention of doctors. In these patients the development of moderate to severe PH, that cannot be explained by the degree of parenchymal lung disease and hypoxia has been defined out-of-proportion PH, and an arbitrary value of PAPm> 35 mm Hg has been established to identify these patients. However, the use of the term out of proportion PH is questionable. Indeed, a direct relationship between ventilator impairment and pulmonary haemodinamics has not been documented in COPD yet. Instead severe respiratory PH phenotype seems a more appropriate term, according to recent data based on ASPIRE Registry [13]. In this study 59 patients with COPD and severe pulmonary hypertension had significantly lower carbon monoxide diffusion, less severe airflow obstruction but not significantly different emphysema scores on computed tomography compared to 42 patients with mild–moderate pulmonary hypertension. 1- and 3-year survival for severe pulmonary hypertension, at 70% and 33%, respectively, was inferior to 83% and 55%, respectively, for mild–moderate pulmonary hypertension. Moreover, mixed venous oxygen saturation, carbon monoxide diffusion, World Health Organization functional class and age, but not severity of airflow obstruction, were independent predictors of outcome. Remarkably, improvement in functional class and/or fall in pulmonary vascular resistance > 20% following targeted treatment identified patients with improved survival.

The diagnosis would be pulmonary hypertension rather than pulmonary arterial hypertension; in fact increased pressures are the result of another disease rather than of diseased arteries. Nonetheless, patients can have both idiopathic pulmonary arterial hypertension and interstitial lung disease contemporaneously. An expert on pulmonary hypertension is required to evaluate such patients and make the correct diagnosis. If through the examination with right heart catheterization the specialist evaluates that the pulmonary hypertension is out of proportion with respect to the lung disease, the patient can be treated. In brief, mild lung disease usually does not advance to severe pulmonary hypertension.

### Pulmonary hypertension and COPD

COPD, defined as a persistent and progressive airflow obstruction, is complicated by numerous comorbidities or associated conditions, as also by frequent episodes of acute exacerbation. Generally, alterations are mainly located in the small airways. Pulmonary hypertension due to chronic respiratory disease and/or hypoxemia represents Group 3 of the Dana Point (2009) clinical classification of PH [14]. This group includes forms of PH due to COPD, pulmonary fibrosis and/or combined pulmonary diseases, in other words chronic infections in which signs of emphysema are visible in the upper lobes and fibrosis in the lower. PH due to respiratory diseases is considered a hemodynamic alteration associated to, and a consequence of, the primary disease, as distinct from the idiopathic form that represents the true disease in which the pulmonary vessels are involved from the very outset.

In terms of definition it is important to underline the meaning of ‘out of proportion’. Cut-off values > 35 and/or > 40 mmHg of PAPm are employed in the literature to define out-of-proportion respiratory PH. This definition was used in the recent guidelines of the European Respiratory Society/European Society of Cardiology [15]. Numerous studies have documented the clinical impact of PH on mortality in COPD patients. Although there is not unanimous agreement about the definition of ‘out-of-proportion PH’ , as a general rule it is considered to be any value of PH in COPD patients with mild-to-moderate ventilatory impairment (forced expiratory volume in 1 sec [FEV_1_] > 50% predicted) or a mPAP > 40 mm Hg in the presence of severe ventilatory deficit but without comorbidities. It should also be noted that in COPD patients levels of mPAP between 21 and 24 mm Hg are considered a pre-PH condition and further studies are required to establish the clinical impact of this range. By ‘chronic pulmonary heart’ definition it is meant hypertrophy and dilation of the right ventricle secondary to PH due to chronic pulmonary disease. However, this term is inappropriate in clinical practice both because the observation of right ventricle anomalies varies according to the method used and because peripheral edema, a clinical surrogate of chronic pulmonary heart, could appear in COPD patients with normal or low central venous pressure. Exercise-induced PH could be used in clinical research but at the moment there is no consensus about the definition. In conclusion, at present PH is defined as resting pulmonary pressure > 25 mm Hg, pulmonary capillary wedge pressure < 15 mm Hg, and/or a reduced cardiac output as measured hemodynamically in COPD patients.

### Pathogenesis of PH secondary to COPD

PH associated to COPD is of a pre-capillary type with low or normal cardiac output and is in particular determined by pulmonary vascular remodeling that provokes an increase in the pulmonary vascular resistance (PVR). Characteristically, it has been shown that cigarette smoking causes vascular remodeling even in early phases of the disease, promoting endothelial dysfunction and airways inflammation. Chronic alveolar hypoxia is responsible for the remodeling of the pulmonary arterioles in particular in people living at high altitude; thus, in COPD patients pulmonary vascular remodeling seems to be the leading cause of PH and not hypoxia, since vascular impairments have been described even in the absence of hypoxemia and long term oxygen therapy does not modify the course of PH in COPD. Nonetheless, local inflammation at the level of the small pulmonary arteries associated to hypoxemia can contribute to pulmonary vascular remodeling.

### Hemodynamic characteristics

Generally PH in COPD is pre-capillary at rest, but PH with elevated capillary pressure is frequently observed in COPD patients with associated emphysema and/or in the presence of comorbidities such as left heart dysfunction. The peculiar nature of the right ventricle, that shows capacity to adapt to the rising afterload and therefore a slow progression towards a functional impairment has to be expected. This feature would explain the well-preserved contractility of the right ventricle at rest and in phase of clinical stability as well as the slow development of PH. In fact in COPD patients and other patients with chronic lung disease, PH is usually of a mild-moderate level: this constitutes an important distinction from the other forms of PH, those of Groups 1 or 4 of the Dana Point classification. Even if PH is mild or moderate at rest, then PAP increases markedly during sleep, physical exercise and, in particular, during exacerbations. Approximately 10-20% of COPD patients at the advanced stage present severe PH with PAP > 35 mm Hg, and this is observed in 3 clinical conditions: a) in patients with severe hypoxemia and hypercapnia associated to severe airflow limitation in whom an increase in resting mPAP can be seen in clinically stable conditions; b) in patients with associated comorbidities such as chronic venous thromboembolism, severe left heart disease or an associated restrictive pulmonary disease; and c) in patients with modest functional limitation associated to severe hypoxemia and hypocapnia. This last condition is defined as out-of-proportion PH. The hemodynamic alterations observed in this form of IP are similar to those observed in the idiopathic form. Recent data from the National Emphysema Treatment Trial indicate that the finding of severe PH at rest is a rare event in emphysema in the absence of comorbidities.

### Clinical impact

The pathogenetic mechanisms underlying dyspnea in COPD patients consist largely in hyperinflation, increased ventilatory demand, muscle weakness and, in more severe patients, hypoxemia and hypercapnia. Nevertheless, the impact of PH on symptoms during exercise is not well established yet, nor it is established if modest increases in mPAP contribute to dyspnea in the course of exercise. In fact, a recent study compared patients with COPD and severe PH with mild-moderate hyperinflation to patients with COPD and mild PH associated to more severe airways limitation who manifested a greater exercise intolerance. More recently it has still been shown that COPD patients with mPAP> 40 mm Hg present a reduced circulatory reserve at end of exercise in contrast to ventilatory reserve exhaustion observed in COPD patients with less severe PH. These findings suggest that lung hemodynamics plays an important role in exercise-related dyspnea in COPD patients with severe PH (mPAP> 40 mm Hg) and not in patients with mild-moderate PH.

### Exercise capacity

Retrospective studies have documented that elevated levels of mPAP are associated to a reduced distance walked in meters on the 6MWT [16] and to a lower maximal power during incremental cardiopulmonary exercise test compared to patients with airways function limitation but mild PH [17]. During the obstructive event the transmural pulmonary arterial pressure is modified in inverse proportion to the level of hypoxia, even if the addition of O_2_ does not modify this condition.

### PH associated to OSA

Pulmonary hypertension has been demonstrated in approximately 17-42% of patients with obstructive sleep apnea syndrome (OSAS) [18,19]. Determining factors in the onset of pulmonary hypertension seem to be PaO_2_, PaCO_2_ and FEV_1_. Studies on animal cell lines have documented that intermittent hypoxia acts as a far more potent stimulus than continuous hypoxia on the activation of multiple transcription factors; among these we find hypoxia-inducible factor-1 (HIF-1), with its second messengers, erythropoietin and vascular endothelial growth factor (VEGF), that together with endothelin-1 (ET-1) are responsible for the structural and pressure modifications [20].

The association of pulmonary hypertension due to hypoventilation and exercise capacity, and the haemodynamic and functional changes, under non-invasive ventilation, has been recently characterized with the presence of severe PH, increase in pro-BNP, and low exercise capacity at baseline, which improved significantly after three months of non invasive ventilation [21]. The PH prevalence estimated in OSA patients is shown in Table 1.

**Table 1:** Prevalence of PH in OSA patients

**Study**	**Sample size**	**PH prevalence (%)**
Schroeder et al. [22]	22	59
Tilkian et al. [23]	12	67
Fletcher et al. [24]	24	79
Podszus et al. [25]	65	20
Weitzenblum [26]	46	20
Krieger et al. [27]	114	19
Sajkov et al. [28]	27	41
Laks et al. [18]	100	42
Chaouat et al. [29]	220	17
Sanner et al. [30]	92	20
Bady et al. [31]	44	27
Sajkov et al. [32]	32	34
Alchanatis et al. [33]	29	21
Arias [34]	23	43
Dumitrascu [35]	45	20

Modified from [36].

### Pulmonary hypertension in obesity hypoventilation syndrome

Obesity places a significant load on the respiratory system, affecting lung volumes, respiratory muscle function, work of breathing, and ventilatory control. Despite this, most morbidly obese individuals maintain eucapnia. However, a subgroup of morbidly obese individuals will develop chronic daytime hypercapnia, described as the Obesity Hypoventilation Syndrome (OHS).

OHS is defined as a disease characterized by:

1. Obesity: BMI ≥ 30 kg/m^2^

2. Chronic alveolar hypoventilation

3. Daytime (awake) hypercapnia (PaCO_2_ ≥ 45 mm Hg)

4. (OSA) sleep respiratory disorders (AHI ≥ 5 with or without hypoventilation during sleep) present in 90% of cases; hypoventilation during sleep (AHI < 5) present in 10% of cases.

The prevalence of estimated PH in OHS is very high, and is between 59 and 88% [37]. Patients with OHS have a lower quality of life with increased health-care expenses and are at a higher risk for the development of pulmonary hypertension and early mortality compared to eucapnic patients with sleep-disordered breathing. Despite the significant morbidity and mortality associated with this syndrome, it is often unrecognized and treatment is frequently delayed.

## Conclusions

Pulmonary hypertension associated to respiratory diseases is a multifactorial disorder which may complicate, and seriously modify, the prognosis of the principal disease. The management and the treatment of this important complication must be evaluated by a team of specialists, including cardiologists, pulmonologists and rheumatologists.

This small review showed the most common disorders associated to pulmonary hypertension, in which the physician has the pivotal role to investigate as soon as possible the presence of this complication, in order to early identify the phenotype associated to the worst outcome.

## Abbreviations

COPD: Chronic obstructive pulmonary disease; IPF: Idiopathic pulmonary fibrosis; OHS: Obesity hypoventilation syndrome; OSA: Obstructive sleep apnea; PH: Pulmonary hypertension; PAH: Pulmonary arterial hypertension.

## Competing interests

The authors declare that they have no competing interests.

## Authors’ contributions

LC conceived and supervised the study, PC designed the study and wrote the manuscript, MS conceived the study, designed the study and wrote the manuscript. All authors read and approved the final manuscript.
